# Repeated Exposure to the “Spice” Cannabinoid JWH-018 Induces Tolerance and Enhances Responsiveness to 5-HT_1A_ Receptor Stimulation in Male Rats

**DOI:** 10.3389/fpsyt.2018.00055

**Published:** 2018-02-27

**Authors:** Joshua S. Elmore, Michael H. Baumann

**Affiliations:** ^1^Designer Drug Research Unit, Intramural Research Program, National Institute on Drug Abuse, National Institutes of Health, Baltimore, MD, United States

**Keywords:** JWH-018, synthetic cannabinoid, serotonin, receptor, spice

## Abstract

Naphthalen-1-yl-(1-pentylindol-3-yl)methanone (JWH-018) is a synthetic compound found in psychoactive “spice” products that activates cannabinoid receptors. Preclinical evidence suggests that exposure to synthetic cannabinoids increases 5-HT_2A/2C_ receptor function in the brain, an effect which might contribute to psychotic symptoms. Here, we hypothesized that repeated exposures to JWH-018 would enhance behavioral responsiveness to the 5-HT_2A/2C_ receptor agonist DOI. Male Sprague-Dawley rats fitted with subcutaneously (sc) temperature transponders received daily injections of JWH-018 (1.0 mg/kg, sc) or its vehicle for seven consecutive days. Body temperature and catalepsy scores were determined at 1, 2, and 4 h post-injection each day. At 1 and 7 days after the final repeated treatment, rats received a challenge injection of either DOI (0.1 mg/kg, sc) or the 5-HT_1A_ receptor agonist 8-OH-DPAT (0.3 mg/kg, sc), then temperature and behavioral responses were assessed. Behaviors induced by DOI included wet dog shakes and back muscle contractions (i.e., skin jerks), while behaviors induced by 8-OH-DPAT included ambulation, forepaw treading, and flat body posture. On the first day of repeated treatment, JWH-018 produced robust hypothermia and catalepsy which lasted up to 4 h, and these effects were significantly blunted by day 7 of treatment. Repeated exposure to JWH-018 did not affect behaviors induced by DOI, but behavioral and hypothermic responses induced by 8-OH-DPAT were significantly augmented 1 day after cessation of JWH-018 treatment. Collectively, our findings show that repeated treatment with JWH-018 produces tolerance to its hypothermic and cataleptic effects, which is accompanied by transient enhancement of 5-HT_1A_ receptor sensitivity *in vivo*.

## Introduction

Synthetic cannabinoids are novel psychoactive substances with pharmacological similarity to the phytocannabinoid Δ^9^-tetrahydrocannabinol (THC), the main psychoactive ingredient in marijuana. Over the last decade, herbal smoking blends consisting of plant material laced with synthetic cannabinoids (i.e., “spice” products) have emerged in the recreational drug marketplace. Analytical investigations of the first spice products revealed that a primary psychoactive component was naphthalen-1-yl-(1-pentylindol-3-yl)methanone, also known as JWH-018 (1, 2). JWH-018 and many of its structural analogs were found in spice products during 2010 through 2013, and JWH-018 is still present on the street today (3, 4). JWH-018 is a potent agonist at cannabinoid type-1 (CB_1_) and cannabinoid type-2 (CB_2_) receptors, which displays at least threefold higher binding affinity than THC at both receptors (5, 6). When administered to mice, JWH-018 produces effects consistent with other CB_1_ receptor agonists, including hypothermia, analgesia, reduced motor activity, and catalepsy (5, 6). In drug discrimination studies, JWH-018 fully substitutes for the THC stimulus cue in both mice and rats (7–9).

Several lines of clinical evidence support a relationship between heavy cannabis use and risk for development of psychosis and schizophrenia (10–13). Although the precise underpinnings of schizophrenia are not fully understood, dysregulation of brain serotonin (5-HT) systems has been implicated in certain psychotic symptoms, such as paranoia and hallucinations (14–16). Preclinical studies in rodents show that exposure to CB_1_ receptor agonists can influence 5-HT receptor responsiveness *in vivo*. For example, Darmani reported that acute pretreatment with various cannabinoids, including THC and the potent cannabinoid receptor agonist (6aR,10aR)-9-(hydroxymethyl)-6,6-dimethyl-3-(2-methyloctan-2-yl)-6H,6aH,7H,10H,10aH-benzo[c]isochromen-1-ol (HU-210), inhibits behavioral effects of the 5-HT_2A/2C_ agonist 2,5-dimethoxy-4-iodoamphetamine (DOI) in mice (17). By contrast, Hill et al. found that repeated treatment with HU-210 for 12 days enhances wet dog shakes induced by DOI in rats (18). Franklin et al. found that 7-day exposure to the cannabinoid agonist 2-[(1R,2R,5R)-5-hydroxy-2-(3-hydroxypropyl) cyclohexyl]-5-(2-methyloctan-2-yl)phenol (CP 55,940) enhances cortisoterone release induced by DOI in rats, and this effect is accompanied by upregulation of 5-HT_2A_ receptors in the hypothalamus (19). Recent evidence suggests a direct interaction between CB_1_ and 5-HT_2A_ receptors in rat brain. In particular, Viñals et al. showed that CB_1_ and 5-HT_2A_ receptor heteromers are present in hippocampus and other brain regions related to memory formation, and these heteromers are necessary for the amnesic effects of THC, but not its analgesic effects (20).

Despite the continued misuse of synthetic cannabinoids by humans, little is known about the functional consequences of repeated administration of JWH-018 or related substances found in spice products. Given the emerging evidence for interactions between cannabinoid and 5-HT systems in the brain, we sought to determine the effects of repeated treatment with JWH-018 on the behavioral responsiveness to selective 5-HT receptor agonists. Specifically, male Sprague-Dawley rats were treated for seven consecutive days with JWH-018, then challenged with DOI or the 5-HT_1A_ receptor agonist 8-hydroxy-2-(dipropylamino)tetralin (8-OH-DPAT) at 1 day and 7 days after the last JWH-018 treatment. Body temperatures and catalepsy scores were determined during the repeated dosing regimen of JWH-018, while body temperatures and agonist-induced behaviors were measured following challenge doses of 5-HT drugs. We hypothesized that repeated exposure to JWH-018 would enhance subsequent behavioral responsiveness to DOI in rats [e.g., see Ref. (18)]. Such increases in 5-HT_2A/2C_ activity produced by cannabinoid exposure could contribute to adverse psychiatric symptoms associated with cannabinoid use.

## Materials and Methods

### Drugs and Reagents

Naphthalen-1-yl-(1-pentylindol-3-yl)methanone (JWH-018) was obtained from Cayman Chemical (Ann Arbor, MI, USA). (−)-2,5-Dimethoxy-4-iodoamphetamine HCl (DOI) and (+)-8- hydroxy-2-(dipropylamino)tetralin HBr (8-OH-DPAT) were obtained from Sigma Aldrich (St. Louis, MO, USA). 5-(4-Chlorophenyl)-1-(2,4-dichlorophenyl)-4-methyl-N-1-piperidinyl-1H-pyrazole-3-carboxamide HCl (rimonabant) was obtained from the pharmacy at the National Institute on Drug Abuse (NIDA), Intramural Research Program (IRP). JWH-018 and rimonabant were dissolved into a 1:1:18 mix of dimethyl sulfoxide:Tween 80:sterile saline, whereas other drugs were dissolved in sterile saline. All injections were administered at a volume of 1.0 mL/kg.

### Animals and Surgery

Male Sprague-Dawley rats (Envigo, Frederick, MD, USA) weighing 250–300 g were double-housed (lights on: 7:00 a.m.–7:00 p.m.) under conditions of controlled temperature (22 ± 2°C) and humidity (45 ± 5%) with free access to food and water. Experiments were performed in accordance with the National Institutes of Health Guide for the Care and Use of Laboratory Animals. Vivarium facilities were fully accredited by the Association for Assessment and Accreditation of Laboratory Animal Care, and study procedures were approved by the NIDA IRP Animal Care and Use Committee. After 2 weeks of acclimation to the vivarium, rats were subjected to surgical procedures and subsequently used for experiments. Rats were rapidly anesthetized with isoflurane using a drop jar which contained a raised floor above a gauze pad saturated with 5 mL of isoflurane. Once fully anesthetized, each rat received a surgically implanted IPTT-300 transponder (Bio Medic Data Systems, Seaford, DE, USA) to facilitate the non-invasive measurement of body temperature via a portable radio frequency reader system (handheld reader). The transponders were 14 mm × 2 mm cylinders implanted subcutaneously (sc) posterior to the shoulder blades *via* a sterile guide needle. Animals were individually housed postoperatively and allowed 7–10 days for recovery.

### Acute JWH-018 Administration and Rimonabant Antagonism

As a first step in our study, we examined the dose–response effects of acute JWH-018 administration in a cohort of 12 rats. Rats were tested once per week for three consecutive weeks. On test day, rats were moved to the testing room in their home cages and given 1 h to acclimate. Feeding trays were removed, and wire lids were placed atop the cages. Rats received sc injections of JWH-018 (0.1, 0.3, or 1.0 mg/kg) or its vehicle. Immediately before injection, and at various times thereafter (0.25, 0.5, 0.75, 1, 1.5, 2, and 4 h post-injection), body temperature was measured using the handheld reader, and animals were observed for 90 s to assess behaviors. Observers were not blind to the drug treatment condition. Rats were assigned a catalepsy score based on three behaviors: immobility (absence of movement), flattened body posture, and splayed limbs (limbs spread out away from the center of the body). Each behavior was given a numerical score of 1 for “behavior absent,” 2 for “behavior present,” or 3 for “behavior continuous/intense”; the three scores were summed to provide a single value ranging from 3 to 9 at each time point.

Once dose–response experiments were completed, we next tested the effect of pretreatment with the CB_1_ receptor antagonist rimonabant on the responses induced by JWH-018 in a cohort of 12 rats. Rats were tested once per week for three consecutive weeks. Rats were pretreated with either 1.0 mg/kg of the CB_1_ receptor antagonist rimonabant or its vehicle 30 min before injection with either 1.0 mg/kg JWH-018 or its vehicle. Body temperature measurements and behavior scoring were carried out as described previously for acute dose–response experiments.

### Repeated Dosing with JWH-018

Results from the acute dose–response experiments demonstrated that 1.0 mg/kg JWH-018 produced robust hypothermia and catalepsy. Thus, this dose was used for the repeated injection experiments carried out in a group of 32 rats. The repeated dosing with JWH-018 or its vehicle was carried out in the vivarium. Rats fitted with surgically implanted sc temperature transponders received a single sc injection of either 1.0 mg/kg JWH-018 or its vehicle, and were returned to their home cages. Immediately before injection, and at 1, 2, and 4 h post-injection, body temperature was measured using the handheld reader, and animals were observed for 90 s. During the observation period, behaviors were scored using the catalepsy scale as detailed above in the Section “Acute JWH-018 Administration and Rimonabant Antagonism.” The JWH-018 injection procedure was repeated daily for seven consecutive days.

### Challenge Injection with Serotonergic Agonists

One day after the last repeated treatment with JWH-018 or vehicle (i.e., day 8, or day 1 of withdrawal), rats were moved to the testing room in their home cages and given 1 h to acclimate. Feeding trays were removed, and wire lids were placed atop the cages. One cohort of 16 rats received 0.1 mg/kg of DOI, whereas another cohort of 16 rats received 0.3 mg/kg of 8-OH-DPAT. The doses of DOI and 8-OH-DPAT were based on preliminary dose–response experiments, which identified drug doses evoking robust behavioral changes that were less than maximal (data not shown). The specific non-contingent behaviors induced by DOI were wet dog shakes and back muscle contractions (i.e., skin jerks). Both behaviors are known to be mediated by 5-HT_2A_ receptors in rats (21–23). The numbers of wet dog shakes and skin jerks present during the observation period were tallied. Wet dog shakes were defined as a rapid and sudden rotation of the head, neck, and shoulders from one side to the other, analogous to the way a wet dog may shake to dry itself. Skin jerks were defined as brief paraspinal muscle contractions of the back muscles in a tail to head direction. Specific non-contingent behaviors induced by 8-OH-DPAT were locomotion in the horizontal plane (i.e., ambulation), forepaw treading, and flattened body posture, components of the 5-HT behavioral syndrome known to be mediated by 5-HT_1A_ receptors (24, 25). Possible scores for each behavior were 0 (behavior absent), 1 (behavior present), or 2 (behavior intense or continuous). At the end of the observation period, the scores for the three behaviors were summed to produce a 5-HT syndrome score for each time point.

After acute serotonergic drug challenge, body temperatures were measured using the handheld reader at 0.25, 0.5, 0.75, 1, 1.25, 1.5, and 2 h post-injection, and behavior scores were given at each time point as appropriate for the treatment received (i.e., wet dog shakes and skin jerks for DOI treatment, and serotonin syndrome scores for 8-OH-DPAT treatment). The acute challenge procedure with DOI and 8-OH-DPAT was repeated 1 week after the last repeated JWH-018 treatment.

### Data Analysis and Statistics

Data were tabulated, analyzed, and graphically depicted using GraphPad Prism (version 5.02; GraphPad Software, Inc., La Jolla, CA, USA). Time-course temperature data were analyzed using a two-way analysis of variance (treatment × time), followed by a Bonferroni *post hoc* test to determine significance between group means at specific time points. Mean temperature data from the DOI and 8-OH-DPAT experiments were evaluated by two-tailed *t*-tests. Catalepsy data from the acute dose–response, rimonabant antagonism and repeated treatments were analyzed by Kruskal–Wallis test (non-parametric), followed by Dunn’s multiple comparison test to determine significance between group means. Summed behavioral score data from the DOI and 8-OH-DPAT challenge experiments were analyzed using a Mann–Whitney test (non-parametric) comparing effects of repeated JWH-018 versus vehicle pretreatments. Statistical analyses were performed on data from all 7 days of the JWH-018 repeated administration experiment, however, Figure 3 only shows data from selected days to make the graphs easier to interpret. *p* < 0.05 was considered the minimal criterion for statistical significance.

## Results

### Effects of Acute JWH-018 Administration

**Figure 1:**
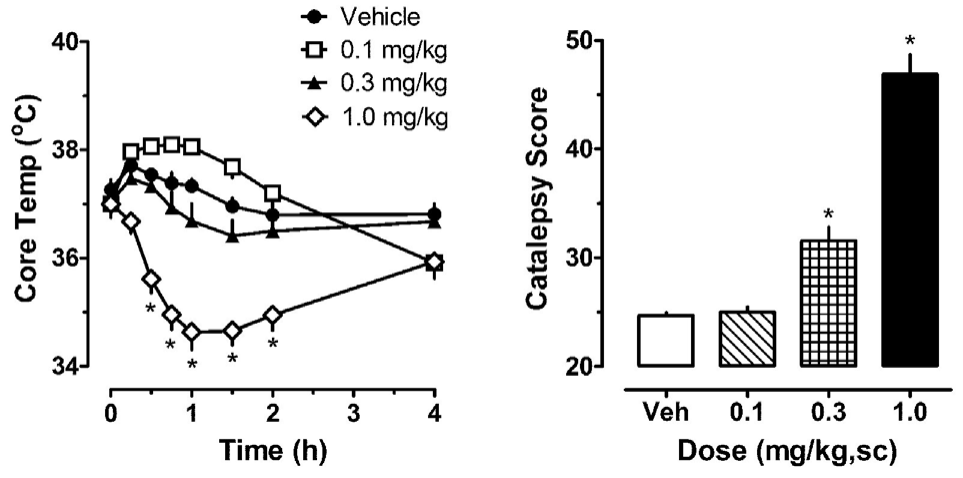
Core temperature measures and summed catalepsy scores for rats receiving acute subcutaneous injections of 0.1, 0.3, and 1.0 mg/kg JWH-018 or its vehicle. Core temperature and behavioral score were recorded at 0, 0.25, 0.5, 0.75, 1, 1.5, 2, and 4 h post-injection, as described in the Section “Acute JWH-018 Administration and Rimonabant Antagonism.” Data are mean ± SEM for *N* = 9 rats per group. *Represents significant effects when compared to the corresponding vehicle-treated group for temperature (Bonferroni, *p* < 0.05) and catalepsy (Dunn’s, *p* < 0.05).

The left panel of Figure 1 illustrates the effect of acute JWH-018 administration on core body temperature in male rats. JWH-018 produced a dose-related change in core temperature (*F*_3,256_ = 111.1, *p* < 0.0001), with significant reductions compared to vehicle control after the 1.0 mg/kg dose at 0.5, 0.75, 1, 1.5, and 2 h post-injection. A maximum decrease of ~3°C was observed at 1 h after the 1.0 mg/kg dose. It is worth noting that 0.1 mg/kg JWH-018 caused a noticeable, albeit non-significant, increase in temperature for the first 2 h, suggesting biphasic dose–response effects of the drug on body temperature. As seen in the right panel of Figure 1, JWH-018 dose-dependently increased the summed catalepsy behavioral score (Kruskal–Wallis statistic 28.53, *p* < 0.0001). Dunn’s test revealed that significant increases from vehicle control were present following the 1.0 mg/kg dose.

**Figure 2:**
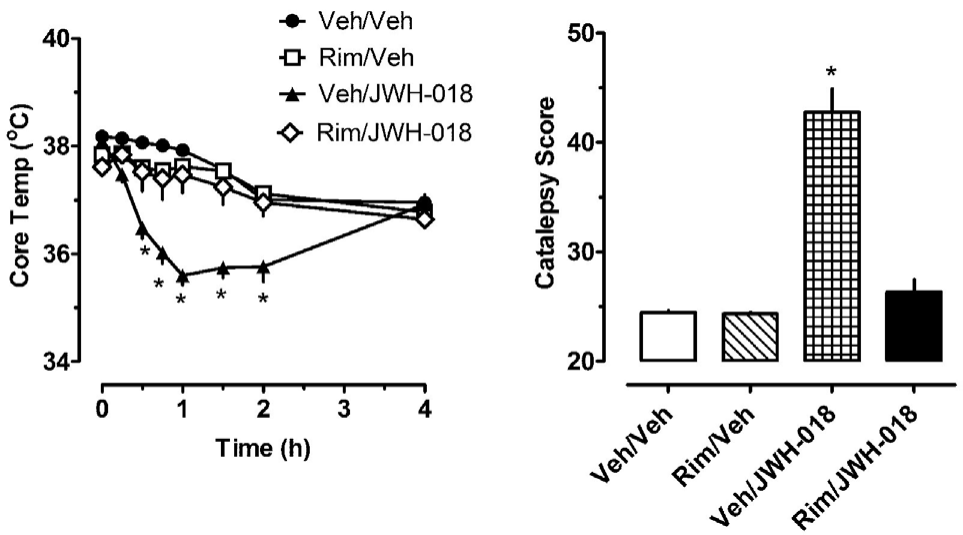
Core temperature measures and summed catalepsy scores for rats receiving either subcutaneous (sc) vehicle (VEH) or 1.0 mg/kg JWH-018 (JWH), 30 min after pretreatment with either sc vehicle (VEH) or 1.0 mg/kg rimonabant (RIM). Core temperature and behavioral score were recorded at 0, 0.25, 0.5, 0.75, 1, 1.5, 2, and 4 h post-injection, as described in the Section “Acute JWH-018 Administration and Rimonabant Antagonism.” Data are mean ± SEM for *N* = 9 rats per group. *Represents significant effects when compared to the corresponding vehicle/vehicle-treated group for temperature (Bonferroni, *p* < 0.05) and catalepsy (Dunn’s, *p* < 0.05).

The left panel of Figure 2 shows that pretreatment with 1.0 mg/kg of the CB_1_ receptor antagonist rimonabant significantly altered the hypothermic effect of 1.0 mg/kg JWH-018 (*F*_3,256_ = 56.79, *p* < 0.0001). Rats treated with rimonabant/JWH-018 were not significantly different from rats treated with vehicle/vehicle, whereas the vehicle/JWH-018 group displayed decreased body temperature that was significantly different from all other groups. Vehicle/JWH-108 rats had significantly decreased body temperature at the 0.5, 0.75, 1, 1.5, and 2 h time points. Likewise, the right panel of Figure 2 shows that rats treated with vehicle/JWH-018 had significantly higher catalepsy scores when compared to all other groups (Kruskal–Wallis statistic 22.32, *p* < 0.0001).

### Effects of Repeated JWH-018 Administration

**Figure 3:**
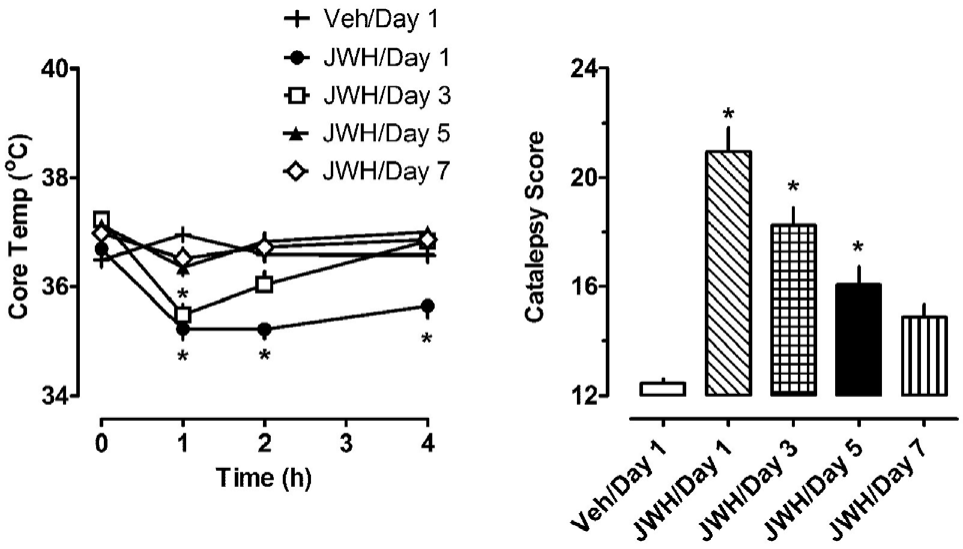
Core temperature measures and summed catalepsy scores for rats receiving either subcutaneous vehicle (VEH) or 1.0 mg/kg JWH-018 (JWH) once daily for seven consecutive days. Core temperature and behavioral score were recorded at 0, 1, 2, and 4 h post-injection each day for 7 days, as described in the Section “Repeated dosing with JWH-018.” Data are mean ± SEM for *N* = 9 rats per group. *Represents significant effects when compared to the vehicle group from day 1 of treatment for temperature (Bonferroni, *p* < 0.05) and catalepsy (Dunn’s, *p* < 0.05).

The left panel of Figure 3 depicts the effects of 1.0 mg/kg JWH-018 or its vehicle on body temperature on days 1, 3, 5, and 7 of repeated treatment. Vehicle administration did not significantly alter body temperature from preinjection values on day 1 of treatment, or during the 7-day treatment regimen (*F*_6,420_ = 0.645, NS). Because vehicle administration did not affect body temperature over the course of repeated injections, we compared the effects of JWH-018 treatments across days to those of vehicle treatment on day 1. Using this analysis, JWH-018 caused significant hypothermia when compared to vehicle (*F*_7,480_ = 22.331, *p* < 0.0001), but the temperature responses changed over the course of treatment. On day 1 of JWH-018 exposure, temperature was significantly reduced from vehicle at the 1, 2, and 4 h timepoints. By day 3 of treatment, hypothermia was observed only at the 1 h timepoint, and on days 6 and 7, no reduction in temperature was observed.

The right panel of Figure 3 depicts the effects of 1.0 mg/kg JWH-018 or its vehicle on summed catalepsy scores on days 1, 3, 5, and 7 of repeated treatment. Vehicle administration did not significantly alter summed catalepsy scores on day 1 of treatment, and there was no change in scores for vehicle-treated rats over the 7-day treatment regimen. Since vehicle administration did not change catalepsy scores over the course of treatment, we compared the effects of JWH-018 treatment across days to the effects of vehicle treatment on day 1. Using this analysis, JWH-018 increased catalepsy scores compared to vehicle (Kruskal–Wallis statistic 63.82, *p* < 0.0001), but the response was attenuated over the course of treatment. Dunn’s test demonstrated that summed catalepsy scores were significantly different when compared to vehicle on days 1 through 6, but not on day 7.

### Effects of Serotonergic Challenge with DOI and 8-OH-DPAT

**Figure 4:**
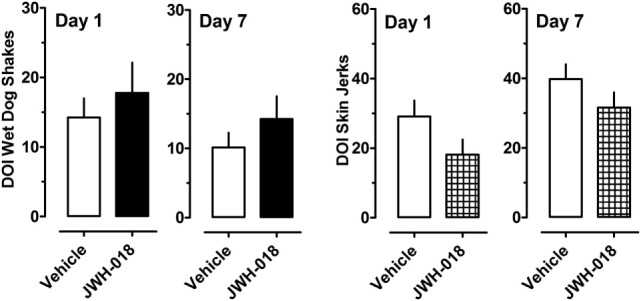
Summed scores for wet dog shakes and back muscle crawls (skin jerks) induced by a subcutaneous challenge injection of 0.1 mg/kg DOI at 1 and 7 days after cessation of repeated JWH-018 treatment. Behavioral scores were recorded at 0, 0.25, 0.5, 0.75, 1, 1.25, 1.5, and 2 h post-injection, as described in the Section “Challenge injection with serotonergic agonists.” Data are mean ± SEM for *N* = 9 rats per group.

Figure 4 depicts the effects of the 5-HT_2A/2C_ receptor agonist DOI on wet dog shakes and skin jerks in rats given the daily regimen of 1.0 mg/kg JWH-018 or its vehicle for 7 days. Rats received 0.1 mg/kg DOI at 1 and 7 days after the last repeated JWH-018 injection. The data demonstrate that there were no significant differences between the pretreatment groups for induction of wet dog shakes (left panel) (Mann–Whitney = 29.50, *p* < 0.833) or skin jerks (right panel) (Mann–Whitney = 18.50, *p* < 0.171) at 1 day after cessation of JWH-018 administration. Similar non-significant effects between pretreatment groups were observed at day 7. DOI did not significantly affect core body temperature in rats pretreated with JWH-018 or vehicle at either test day (data not shown).

**Figure 5:**
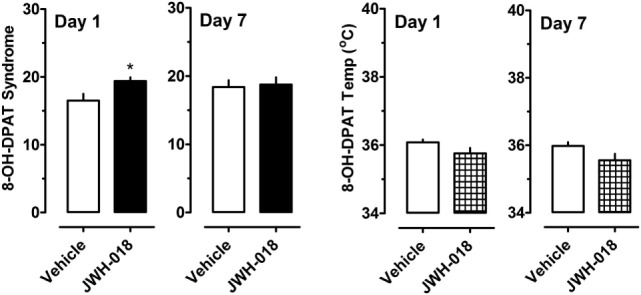
Summed scores for serotonin syndrome behaviors and mean temperature recordings induced by a subcutaneous challenge injection of 0.3 mg/kg 8-OH-DPAT at 1 and 7 days after cessation of repeated JWH-018 treatment. Behavioral scores and core temperatures were recorded at 0, 0.25, 0.5, 0.75, 1, 1.25, 1.5, and 2 h post-injection, as described in the Section “Challenge injection with serotonergic agonists.” Data are mean ± SEM for *N* = 9 rats per group. *Represents significant effects when compared to the group that received repeated vehicle treatment (Mann–Whitney, *p* < 0.05).

A separate cohort of rats was given 0.3 mg/kg of the 5-HT_1A_ agonist 8-OH-DPAT at 1 and 7 days after the daily regimen of 1.0 mg/kg JWH-018 or its vehicle. Specific behaviors and hypothermic responses to 8-OH-DPAT were measured. The left panel of Figure 5 shows that there was a small yet significant enhancement of 5-HT syndrome score for the JWH-018 pretreated animals at 1 day after the last repeated treatment (Mann–Whitney = 9.50, *p* < 0.019), but this effect disappeared at 7 days (Mann–Whitney = 29.00, *p* < 0.791). The right panel of Figure 5 shows that mean hypothermic responses produced by 8-OH-DPAT were slightly lower in JWH-018 pretreated rats, but this effect was not significantly different between pretreatment groups day 1 (*t* = 1.854, df = 14, *p* < 0.085) or at day 7 (*t* = 1.925, df = 14, *p* < 0.075).

**Figure 6:**
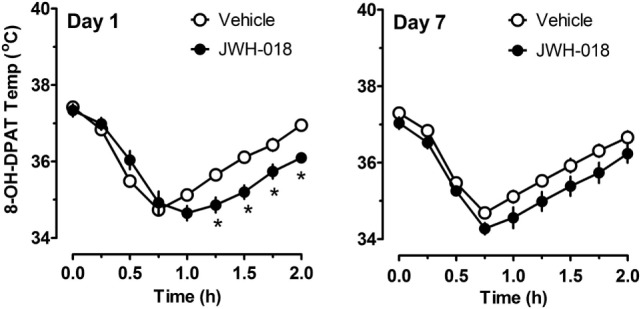
Time-course of core body temperature changes induced by a subcutaneous challenge injection of 0.3 mg/kg 8-OH-DPAT at 1 and 7 days after cessation of repeated JWH-018 treatment. Temperatures were recorded at 0, 0.25, 0.5, 0.75, 1, 1.25, 1.5, and 2 h post-injection, as described in the Section “Challenge injection with serotonergic agonists.” Data are mean ± SEM for N = 9 rats per group. *Represents significant effects when compared to vehicle-pretreatment group at specific time points (Bonferroni, *p* < 0.05).

Because there were trends for enhanced hypothermic responses to 8-OH-DPAT in rats pretreated with JWH-018 (e.g., *p* < 0.07), we evaluated the raw time-course data for temperature responses in this experiment. Figure 6 illustrates that rats exposed to JWH-018 displayed enhanced hypothermic responses to 8-OH-DPAT when compared to vehicle-pretreated rats at day 1 after cessation of repeated treatments (*F*_1,126_ = 17.74, *p* < 0.001). *Post hoc* tests revealed that temperature was significantly decreased in the JWH-018 group compared to the vehicle group at 1.25, 1.5, 1.75, and 2 h time points after injection of 8-OH-DPAT. The enhanced responsiveness to 8-OH-DPAT in the JWH-018 group was still evident at 7 days after cessation of treatment (*F*_1,126_ = 23.26 *p* < 0.001), though *post hoc* tests found no differences between pretreatment groups at any time point.

## Discussion

The psychiatric literature supports a strong relationship between heavy cannabis use and risk for subsequent psychosis and schizophrenia (12). In addition, misuse of synthetic cannabinoids such as JWH-018 and its analogs is associated with induction of more severe psychotic symptoms when compared to the effects of marijuana (26, 27). Previous studies in rats demonstrate that exposure to synthetic cannabinoids can induce enhanced sensitivity to 5-HT_2A/2C_ receptor activation (18) and upregulation of 5-HT_2A/2C_ receptors in specific brain regions (28, 29). The aim of the present study was to use the popular synthetic cannabinoid JWH-018 to further explore the relationship between repeated cannabinoid exposure and serotonergic dysregulation. JWH-018 is a potent non-selective cannabinoid receptor agonist that was found in the first generation of spice products (1, 2). The present experiments yielded three primary findings. First, in contrast to the results of others [e.g., see Ref. (18)], we detected no significant difference in responsiveness to the 5-HT_2A/2C_ receptor agonist DOI between rats pretreated with synthetic cannabinoids compared to those pretreated with vehicle. Second, we found a modest and significant enhancement of sensitivity to behavioral and hypothermic effects induced by 8-OH-DPAT in rats exposed to repeated injections of JWH-018. Finally, our data show that rats receiving daily injections of JWH-018 develop profound tolerance to its hypothermic and cataleptic effects, such that these effects are nearly absent after 7 days of treatment.

In our experiments, male rats were subjected to seven consecutive days of JWH-018 injections, then given a challenge dose of either the 5-HT_2A/2C_ agonist DOI or the 5-HT_1A_ agonist 8-OH-DPAT at 1 and 7 days after cessation of the repeated dosing regimen. Typical behavioral responses to DOI administration in rats are wet dog shakes (analogous to the head twitch response in mice) and back muscle contractions, also known as skin jerks (21–23). These responses are accepted as specific indicators of 5-HT_2A_ receptor activation since the effects are blocked by selective 5-HT_2A_ receptor antagonists. We found no significant difference in the number of wet dog shakes or skin jerks induced by DOI between the cannabinoid-treated and vehicle-treated groups at either time point. Our findings differ from those of Hill et al., who reported that 12 days of HU-210 administration in rats increases DOI-induced wet dog shakes but decreases skin jerks (18). It is noteworthy that we observed trends for augmented wet dog shakes and attenuated skin jerks in rats exposed to JWH-018, but these effects did not reach significance, perhaps due to variability in the behavioral data. We also administered a submaximal dose of 0.1 mg/kg DOI for our experiments, whereas Hill et al. administered a 10-fold higher dose. Hill et al. theorized that the differential effects of HU-210 on the two behaviors induced by DOI could be due to region-specific changes in 5-HT_2A_ receptors caused by the cannabinoids. This hypothesis was later supported by the work of Franklin et al., who found that 7-day administration of CP 55,940 increased DOI-induced prolactin release, while producing no change in brain levels of 5-HT_2A_ receptor mRNA (19). It is well known that HU-210 displays a much longer time course of action when compared to other synthetic cannabinoids, including JWH-018, and may bind pseudo-irreversibly to the CB_1_ receptor. Hruba and McMahon found that rhesus monkeys trained to discriminate THC from vehicle continued to emit drug-appropriate responses for 48 h after administration of HU-210, while such responses to THC and CP 55,940 ceased after 5 h. The same study found that rimonabant treatment increased the ED_50_ values of THC and CP 55,940 discrimination by 12.5 fold, while only causing a 3.8-fold increase for HU-210 (30). Thus, the discrepancies between our results and those of Hill et al. could be due to the use of different cannabinoid agonists for the repeated treatment regimen.

Darmani administered a range of doses of THC, HU-210 and CP 55,940 to mice, followed by an injection of DOI 20 min later, and found that the cannabinoids dose-dependently reduce DOI-induced behaviors (17). Our study used a repeated cannabinoid administration paradigm followed by the administration of DOI after 1 and 7 days of withdrawal, so this may help to explain the differences between our results and those of Darmani. The present findings in rats show that administration of CB_1_ agonists causes considerable catalepsy (see Figures 1–3), so it seems possible that suppression of motor activity caused by acute cannabinoids could influence subsequent behavioral effects of 5-HT_2A_ receptor agonists. We purposefully designed our experiments to examine the responsiveness to 5-HT agonists at 1 and 7 days after the acute effects of cannabinoid administration had subsided.

We found a modest yet significant increase in the behavioral and hypothermic effects induced by 8-OH-DPAT in rats receiving repeated JWH-018 treatments when compared to those receiving repeated vehicle treatments. The augmented sensitivity to 8-OH-DPAT resolved by 7 days after cannabinoid exposure. In a previous study, Hill et al. found that repeated injections of HU-210 for 12 consecutive days reduce the hypothermic and corticosterone responses produced by 8-OH-DPAT in vehicle-treated animals (18). Both hypothermia and corticosterone release are presumably mediated by 5-HT_1A_ receptors in the brain (31), thus Hill et al. found that repeated administration of HU-210 decreases 5-HT_1A_ activity in response to agonism, whereas we found the exact opposite in rats exposed to JWH-018. It seems possible that discrepancies between our results and those of Hill et al. could be due to the use of different cannabinoid agonists, as noted above. On the other hand, Zavitsanou et al. demonstrated that repeated injections of HU-210 increase 5-HT_1A_ receptor density and mRNA levels in the hippocampus and amygdala of male rats (32), a finding consistent with the possibility of enhanced 5-HT_1A_ receptor responsiveness after cannabinoid exposure. Our data demonstrating an increase in 5-HT_1A_ receptor sensitivity after exposure to JWH-018 is a unique finding, and its relationship to the development of psychiatric symptoms following cannabinoid exposure warrants further study. Future research should determine whether 5-HT_1A_ upregulation occurs after repeated exposure to other synthetic cannabinoids. Importantly, and in contrast to existing findings using other cannabinoid compounds, our data show that repeated exposure to JWH-018 does not induce robust alterations in 5-HT_2A_ receptor responsiveness, but increases 5-HT_1A_ responsiveness.

In addition to assessing changes in serotonergic activity after cannabinoid exposure, one of the secondary aims of our study was to examine pharmacological responses to repeated JWH-018 injections. Rats in our study had implantable temperature transponders to facilitate the non-invasive measurement of body temperature. JWH-018 was shown to dose-dependently cause hypothermia and catalepsy, both of which were reversed by rimonabant (see Figure 2). The present data showing acute decreases in body temperature after JWH-018 administration in rats are consistent with previous findings from our laboratory and others, which show dose-related hypothermic effects of JWH-018 as assessed by radiotelemetry or rectal probes to measure core temperatures (33–36). As the repeated injection procedure progressed in our study, rats began to develop tolerance to both the hypothermic and cataleptic effects produced by JWH-018. By day 5 of repeated treatments, the effects of JWH-018 became submaximal at all time points, and continued to decrease in the two remaining days. By day 7 of repeated treatments, the temperature and cataleptic effects JWH-018 were not significantly different from vehicle-treated animals. Previous studies in mice have shown that repeated daily injections of THC or synthetic cannabinoids produce behavioral tolerance due to downregulation and desensitization of CB_1_ receptors (37). Likewise, acute JWH-018 is reported to induce downregulation of CB_1_ receptors in cultured neurons by a mechanism involving rapid receptor internalization (38).

The experiments of Tai et al. showed that mice develop tolerance to the hypothermic effects of JWH-018, but not the locomotor suppressing effects (39). The apparently contradictory findings between our results and those of Tai et al. may be due to species-specific differences between rats and mice. Tai et al. also showed that mice repeatedly exposed to THC develop a cross tolerance to the effects of JWH-018. The development of tolerance to cannabis is well documented, and the demonstration of tolerance to JWH-018 could have important clinical implications (40, 41). Dose escalation in human THC users is often observed as a means to overcome cannabis tolerance, but this phenomenon likely will not cause acute bodily harm. By contrast, dose escalation with JWH-018 or other potent synthetic cannabinoids could be more dangerous. Typical adverse effects arising from synthetic cannabinoid use are tachycardia, agitation, and nausea; more serious adverse events include seizures, acute kidney injury, new onset psychosis, severe cardiac crisis, and death (27, 42). Further research is required to determine if such dose escalation occurs in humans who use synthetic cannabinoids.

To summarize, we found that repeated treatment with the synthetic cannabinoid JWH-018 does not lead to significant changes in 5-HT_2A_ receptor responsiveness in rats, but produces transient increases in 5-HT_1A_ receptor responsiveness. These findings, unlike data generated using other synthetic cannabinoids, do not support the contention that exposure to cannabinoid receptor agonists universally leads to an increase in 5-HT_2A_ receptor responsiveness, suggesting that alteration of 5-HT_2A_ neurotransmission may not be responsible for the link between cannabinoid exposure and the subsequent development of psychotic symptoms. On the other hand, rats in our experiments developed tolerance to both hypothermia and catalepsy produced by JWH-018 after several consecutive days of treatment, findings which differ from prior work in mice suggesting that tolerance only develops to hypothermic effects. Synthetic cannabinoid tolerance in humans could potentially lead to dose escalation, which could be more dangerous with synthetic cannabinoids when compared to marijuana.

## Ethics Statement

Experiments were performed in accordance with the National Institutes of Health Guide for the Care and Use of Laboratory Animals. Vivarium facilities were fully accredited by the Association for Assessment and Accreditation of Laboratory Animal Care, and study procedures were approved by the NIDA Intramural Research Program Animal Care and Use Committee.

## Author Contributions

JE and MB were responsible for experiment design, statistical analysis, and manuscript writing. JE collected the data.

## Conflict of Interest Statement

The authors declare that the research was conducted in the absence of any commercial or financial relationships that could be construed as a potential conflict of interest.
